# A Novel Anticancer Peptide Derived from *Bryopsis plumosa* Regulates Proliferation and Invasion in Non-Small Cell Lung Cancer Cells

**DOI:** 10.3390/md21120607

**Published:** 2023-11-24

**Authors:** Heabin Kim, Hyun-Taek Kim, Seung-Hyun Jung, Jong Won Han, Seonmi Jo, In-Gyu Kim, Rae-Kwon Kim, Yeon-Jee Kahm, Tae-Ik Choi, Cheol-Hee Kim, Jei Ha Lee

**Affiliations:** 1 Department of Genetic Resources, National Marine Biodiversity Institute of Korea, Seocheon 33662, Republic of Korea; khb7116@mabik.re.kr (H.K.); zebrajung@mabik.re.kr (S.-H.J.); jwhan@mabik.re.kr (J.W.H.); joseonmi@mabik.re.kr (S.J.); 2 Soonchunhyang Institute of Medi-Bio Science (SIMS), Soonchunhyang University, Cheonan-si 31151, Republic of Korea; hyun-taek.kim@sch.ac.kr; 3 Department of Radiation Biology, Environmental Safety Assessment Research Division, Korea Atomic Energy Research Institute, Daejeon 34057, Republic of Korea; igkim@kaeri.re.kr (I.-G.K.); rkim@kaeri.re.kr (R.-K.K.); kahmyj@kaeri.re.kr (Y.-J.K.); 4 Department of Radiation Science and Technology, Korea University of Science and Technology, Daejeon 34113, Republic of Korea; 5 Department of Biology, Chungnam National University, Yuseong-gu, Daejeon 34134, Republic of Korea; c860523@naver.com (T.-I.C.); zebrakim@cnu.ac.kr (C.-H.K.)

**Keywords:** *Bryopsis plumose*, anticancer peptide, EMT, NSCLC, cancer

## Abstract

The discovery of new highly effective anticancer drugs with few side effects is a challenge for drug development research. Natural or synthetic anticancer peptides (ACPs) represent a new generation of anticancer agents with high selectivity and specificity. The rapid emergence of chemoradiation-resistant lung cancer has necessitated the discovery of novel anticancer agents as alternatives to conventional therapeutics. In this study, we synthesized a peptide containing 22 amino acids and characterized it as a novel ACP (MP06) derived from green sea algae, *Bryopsis plumosa*. Using the ACP database, MP06 was predicted to possess an alpha-helical secondary structure and functionality. The anti-proliferative and apoptotic effects of the MP06, determined using the cytotoxicity assay and Annexin V/propidium iodide staining kit, were significantly higher in non-small-cell lung cancer (NSCLC) cells than in non-cancerous lung cells. We confirmed that MP06 suppressed cellular migration and invasion and inhibited the expression of N-cadherin and vimentin, the markers of epithelial–mesenchymal transition. Moreover, MP06 effectively reduced the metastasis of tumor xenografts in zebrafish embryos. In conclusion, we suggest considering MP06 as a novel candidate for the development of new anticancer drugs functioning via the ERK signaling pathway.

## 1. Introduction

Lung cancer-associated mortality is more common than that caused by breast, prostate, colorectal, or brain cancers. Non-small cell lung cancer (NSCLC) is the most major subtype (<80%) of lung cancer; lung adenocarcinoma and lung squamous cell carcinoma are the most common NSCLCs [1,2]. Patients with NSCLC have a poor prognosis and are diagnosed at the metastatic stage of disease [3]. Malignant cells such as NSCLC cells often undergo epithelial–mesenchymal transition (EMT), a developmental program crucial for embryogenesis, tissue remodeling, and wound healing [4,5]. During tumor progression, cancer cells undergo specific changes to acquire mesenchymal phenotype; they lose their polarity and cell–cell adhesion; thereby, migratory and stem-like cells are generated. Mesenchymal cells can escape from the primary tumor and reach distant sites, leading to the poor prognosis in lung adenocarcinoma [6]. Tumor metastasis involves various cellular processes, such as local invasion, intravasation, transport, extravasation, and colonization, leading to dissemination and adaptation. EMT plays a key role in promoting cancer metastasis. During EMT, cancer cells overexpress mesenchymal-related proteins, such as N-cadherin, vimentin, and matrix metallo-proteases (MMP), which enable them to migrate [7,8]. Vimentin, a hallmark of mesenchymal-like conversion of epithelial cells, is overexpressed in NSCLC, which is associated with poor prognosis [9]. In contrast, high levels of E-cadherin diminish the number of cancer stem cells and decrease xenograft tumor growth in A549 cells [10]. Considering the role of EMT in metastatic progression, controlling EMT is considered a strategy for suppressing metastasis and improving the quality of life of patients [11].

Currently, therapeutic approaches to lung cancer include surgical tumor removal, platinum-based chemotherapy, targeted therapy for molecular changes, and radio- and immune-therapies. Despite the advances in current therapies, their adverse effects can reduce the quality of life and induce life-threatening hypersensitivity reactions in patients. NSCLC frequently relapses owing to oncogenic driver mutations involved in the evasion to the immune system, resistance of therapy, metastasis, and activated invasion [12,13,14]. Moreover, most anticancer drugs cannot selectively target cancer cells without damaging healthy tissues, which results in toxicity and adverse side effects [15]. Among the existing clinical treatments for cancers, combined therapies can effectively achieve better overall survival and cure than that offered by a single therapy [16]. Therefore, further research is required to develop novel alternative therapeutic methods or anticancer drugs with high selectivity for cancer cells and low toxicity and propensity for drug resistance.

Anticancer peptides (ACPs) have emerged as alternatives to conventional anticancer drugs [17]. The small size (5–50 amino acids) and high solubility of ACPs provide better pharmacokinetics, higher uptake in target tissues, and suitability for the rapid removal from non-target tissues than those of other existing therapeutic agents, facilitating the anti-cancer therapy [18]. *Bryopsis plumosa* is one of the common marine green algae, producing a number of active components with a variety of functional structures and biological properties, such as polyphenols, peptides, and polysaccharides. These biological components have shown potential effects as interesting textural, gelling, antioxidant, and antimicrobial properties, as well as anticancer activity [19,20,21]. Here, we discover a novel ACP(MP06) derived from green sea algae, *Bryopsis plumosa,* exhibiting selective anticancer activity against lung cancer cells and minimal toxicity in human lung fibroblasts, which suggests that MP06 can regulate the remodeling of the cytoskeleton and inhibit EMT-related transcription factors, consequently leading to the downregulation of EMT in NSCLC cells. Therefore, inhibition of cell adhesion, migration, and invasion suppressed tumor growth and progression; moreover, cell migration was significantly inhibited in zebrafish xenografted with NSCLC cells. These results provide new insights into the mechanism underlying the inhibition of tumor migration through the reversal of EMT via the ERK signal pathway [22].

## 2. Results

### 2.1. Designing, Synthesis, and Characteristics of MP06

**Figure 1:**
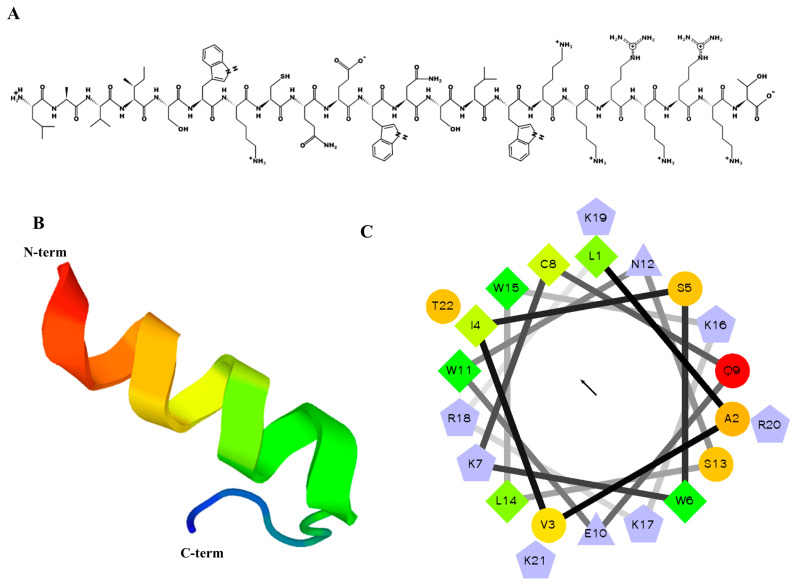
Predicted helical secondary and three-dimensional structures of MP06 anticancer peptide. (**A**) Two-dimensional drawing of the peptide using the PepDraw software (https://pepdraw.com, accessed on 12 April 2022) (**B**) The predicted three-dimensional secondary structure of MP06 from PEP-FOLD3. (**C**) Helical wheel plots of MP06 sequences showing amphipathicity quantified as hydrophobic moments. Shows hydrophilic residues as circles, hydrophobic residues as diamonds, and potentially positively/negatively charged as triangles/pentagons. The most hydrophobic residue is green, and the amount of green decreases proportionally to the hydrophobicity, with zero hydrophobicity coded as yellow. Hydrophilic residues are coded red, with pure red being the most hydrophilic (uncharged) residue and the amount of red decreasing proportionally to the hydrophilicity. The potentially charged residues are light blue. The arrow in the middle of the circle indicates the hydrophobic direction formed by amino acids with high hydrophobicity.

For screening of ACPs from *Bryopsis plumosa*, cDNA sequences were cut off below 200 bp (<60 amino acids) to remove long sequences, and peptide sequences containing 60 amino acids or less were analyzed. The peptides for developing anticancer drugs were screened based on the prediction of ACP tools, such as CancerPPD (http://crdd.osdd.net/raghava/cancerppd, accessed on 12 April 2022) and AntiCP 2.0 (http://webs.iiitd.edu.in/raghave/anticp2, accessed on 12 April 2022), which generated sequence information on po-tent ACP. Similarity analysis of the selected peptide sequence was performed using a self-constructed anti-cancer peptide database [23,24]. The presence of a 22 amino acid sequence (LAVISWKCQEWNSLWKKRKRKT) in anti-cancer peptides was verified using the ACP model. The predicted alpha-helix secondary and three-dimensional structures of MP06 are depicted in Figure 1. The peptide exhibited a total net charge of +6 and hydrophobicity of 22.07 kcal/mol. MP06 possesses lysine and/or arginine residues, which of positive charge can interact with the negatively charged cancer cell membranes. The molecular weight of the synthesized MP06, with 92.5% purity, was 2788 Da (Supplementary Figure S1). Although we synthesized 21 ACPs predicted using the database, only MP06 exhibited anticancer activity (Supplementary Figure S2, Table S1). It can be assumed that different sequences have specific cytotoxic effects based on their properties and structures.

### 2.2. Cellular Viability in Lung Cancer Cells

**Figure 2:**
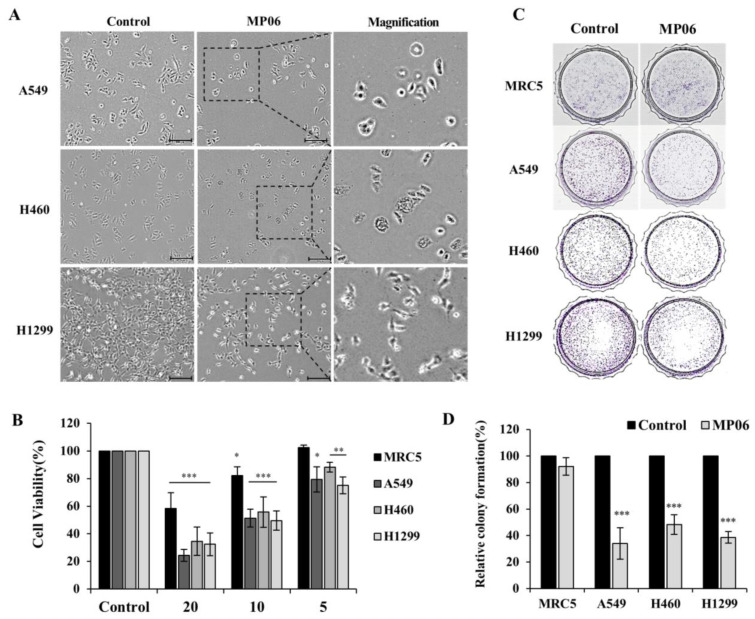
Investigating the role of MP06 anticancer peptide in NSCLCs. (**A**) Morphologic changes of A549, H460 and H1299 cells treated with 10 μM MP06 compared water-treated control cells. (**B**) Effect of MP06 at a concentration of 20, 10 and 5 μM on the growth of normal lung fibroblast and NSCLCs for 48 h. (**C**) The difference in colony forming ability on treatment of 10 μM MP06 in lung cells. (**D**) Quantification of relative colony formation in NSCLCs treated with MP06. Scale bars: 200 µm. * *p* < 0.05, ** *p* < 0.01 and *** *p* < 0.001.

To study the effect of the peptide MP06 on NSCLC, three cell lines were selected. These cells (A549, H460, and H1299) are widely used for research and drug discovery of lung cancer. In NSCLC cells, 10 μM MP06 induced morphological changes involved in the typical EMT and apoptosis. Significant morphological changes from a spindle shape to a cobblestone-like shape were detected in lung cancer cells treated with MP06 (Figure 2A). CCK-8 assay revealed the cytotoxicity of synthetic MP06 against cancer and non-cancerous cells (Figure 2B). The results indicate a marked difference in the cytotoxicity of 5–20 μM MP06 between NSCLC cells and human fetal lung fibroblasts (MRC5). MP06 was considerably more toxic to cancer cells than to the fibroblasts, which reflects its selective anti-cancer activity. The colony formation assay, performed to determine the association of MP06 with tumor growth, revealed that MP06 treated cells exhibit significantly reduced growth compared to that of the water-treated control cells (Figure 2C). These results indicate that MP06 is considerably more toxic to cancer cells than to normal lung cells and demonstrates selective anticancer activities.

### 2.3. Effect of MP06 on EMT

**Figure 3:**
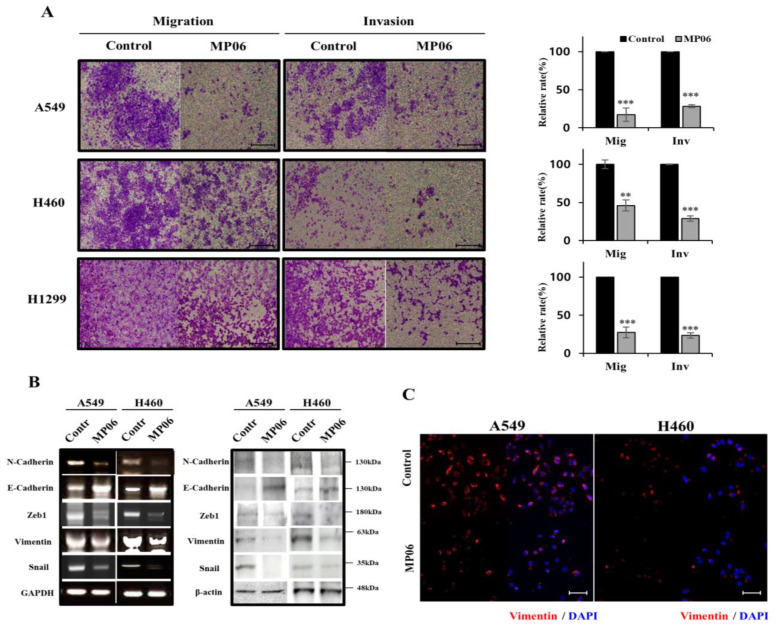
Regulation of epithelial–mesenchymal transition (EMT) in NSCLCs by MP06. (**A**) Influence MP06 on NSCLCs migration (24 h) and invasion (48 h). Scale bars: 200 µm. (**B**) Expression level of epithelial–mesenchymal transition (EMT) markers with 10 μM MP06 compared water-treated control cells (contr) in NSCLCs for 48 h using RT-PCR and Western blotting assay. GAPDH and β-actin were used as housekeeping genes on the basis of their consistency of expression. (**C**) The expression of vimentin in A549 and H460 cells treated with MP06 using Immunofluorescence (IF) analysis. Scale bars: 100 µm. ** *p* < 0.01 and *** *p* < 0.001.

The EMT is correlated with the profound morphological and physiological changes that occur in several cancers. As EMT is associated with therapeutic resistance and recurrent cancer, effectively controlling the EMT-related cell signal pathways may prevent cancer metastasis [25,26]. To test whether MP06 regulates the migration and invasion of cancer cells, we evaluated the migration and invasion assay (Figure 3A). The migration/invasion assay showed that the treatment of MP06 significantly suppressed migration and invasion in NSCLCs. The expression of EMT regulatory markers, including N-cadherin, E-cadherin, and vimentin, and transcription factors Zeb1 and Snail were detected in MP06-exposed cells using RT-PCR and Western blotting (Figure 3B). The result showed an increase in E-cadherin expression and decreased N-cadherin, Zeb1, snail and vimentin, which is consistent with the results of the immunofluorescence analysis of vimentin expression (Figure 3C). The results revealed that MP06 significantly reduced the invasive and migratory capacities of NSCLC cells.

### 2.4. Effect of MP06 on Apoptosis and Signaling in NSCLC Line

**Figure 4:**
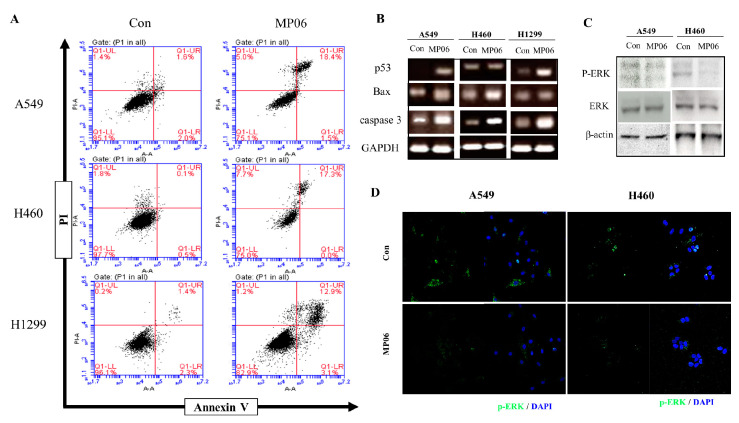
The effect of MP06 on apoptosis of NSCLCs. (**A**) Detection of apoptosis and necrosis in NSCLCs via Annexin V/PI apoptosis kit staining. (**B**) Expression of apoptosis-related genes such as P53, Bax, and caspase3 in NSCLCs treated with 10 μM MP06. (**C**) The expression of phosphorylation ERK in MP06 treated A549 and H460 cells using Western blotting assay. (**D**) The expression of p-ERK in A549 and H460 cells treated with MP06 using IF analysis. Scale bars: 100 µm.

We proceeded to elucidate cell death relative to ACP in NSCLCs, which were stained with Annexin V-FITC and a propidium iodide apoptosis kit. Flow cytometric analysis revealed apoptosis induced after treatment using MP06 (10 μM) for 48 h. The proportion of apoptotic cells increased by 18.4%, 17.3%, and 12.9% in A549, H460 cells, and H1299 cells, respectively, compared with that in the control group (Figure 4A). To investigate the mechanisms underlying the enhanced pro-apoptotic effect of MP06, the cellular expression of apoptosis related markers, such as p53, Bax, and caspase-3, was estimated using RT-PCR. The result presented an increase in apoptosis markers, which is consistent with apoptosis analysis using FACS (Figure 3B). These results suggest that MP06 induces cell death (apoptosis) in lung cancer cells.

Considering that the signaling pathway occurs through ERK phosphorylation, we investigated the expression of cellular level using immunoprecipitation and immunofluorescence. The results showed that phosphorylated ERK were reduced after MP06 treatment in both A549 and H460 cells (Figure 4C,D). Therefore, we suggest that MP06 induced anti-metastatic and apoptotic effects contribute to the inhibition of ERK signaling in NSCLC cells.

### 2.5. Effect of MP06 in Zebrafish Xenograft

**Figure 5:**
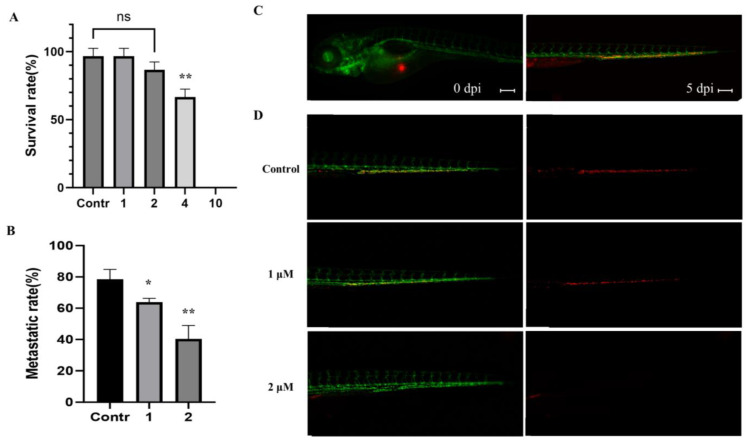
Inhibition of metastasis on A549 cells with MP06 in zebrafish embryos (**A**) Survival evolution through time of 48 h post fertilization (hpf) zebrafish embryos exposed to MP06 at different concentrations. (**B**) Quantification of metastatic rate of Tg(*kdrl*:GFP) zebrafish embryos microinjected with CM-DiI A549 cells at 5 dpi in zebrafish xenograft treated with MP06 (**C**) Injected A549 cells (red) immediately after xenograft (left) and at 5 dpi (right) (**D**) Xenografted zebrafish treated with 1, 2 μM MP06. Representative images show the invasive A549 cells (red) in the tail region of the embryos via vessels (green). (n = 30, ns: not statistically significant, Scale bars: 200 µm. * *p* < 0.01, ** *p* < 0.001).

To demonstrate the selectivity of MP06, an antitumor agent, for xenografted cancer cells in zebrafish, we investigated its toxicity at different concentrations in the zebrafish embryos. Ten embryos of zebrafish were selected per batch, and each batch was exposed to 1, 2, 4, and 10 μM MP06. In Figure 5A, MP06 at concentrations > 2 μM significantly reduced the survival rate in zebrafish embryos. All zebrafish died after treatment with 10 μM MP06, which may have potential toxicity depending on the concentration in zebrafish models compared to in vitro. The toxicity of MP06 was assessed in terms of the developmental rates of embryos and determined the concentration of MP06 to treatment. (Figure 5A).

We conducted a zebrafish xenograft experiment following a previous study on cancer invasion [27]. To obtain the optimal visualization, we used the Tg(*kdrl*:GFP) transgenic zebrafish line, which expresses green fluorescent protein to exhibit a green fluorescent vasculature. CM-DiI dye labeled A549 cells were injected into the yolk sac region of the embryos via vessels under a microscope. Cells were disseminated within the fish body at 5 days post-injection (dpi) using confocal microscopy, where the spread of cancer cells was quantified to determine metastasis in the tail (Figure 5B). The stained A549 cells, which are invasive adenocarcinoma, metastasized to spread at the fishtail consistently in vivo (Figure 5C). In contrast, the cells treated with MP06 showed low metastatic potential in zebrafish models (Figure 5D). These results confirmed that the zebrafish xenograft model can be used for functional analysis of ACP and its effect on A549 cells.

## 3. Discussion

The discovery of new therapeutic uses of existing drugs is a valuable approach for treating cancers due to their low toxicity and in vivo tolerability. Previous findings indicating ACP-mediated regulation of proliferation and invasion in cancer cells have prompted clinical trials for evaluating the impact of ACP in patients with lung cancer. The expected ACPs showed low toxicity in cancer cells and good solubility in distilled water. ACP has emerged as an attractive target for the development of cancer therapies and several ACP drugs have been investigated through trials [28]. ACPs possess cationic residues, such as lysine (K) and arginine (R), positioned at the end of the sequence [29,30]. MP06 contains six consecutive amino acid residues, K and R, which are present at the C-terminus of the sequence, suggesting the importance of cytotoxicity in cancer cell lines for the anti-cancer effect of ACP. The anticancer activity of MP06 can be attributed to the presence of K and R residues in its C-terminus, which supports the interactions with cellular membranes and regulates toxicity [31]. Moreover, the high hydrophobicity of these peptides favors their penetration into the lipid bilayer of the cell membrane. Additionally, a previous report indicated increased cytotoxicity of tryptophan (W) residue-containing peptides against A549 cells; this result is comparable to our findings that demonstrate three tryptophan amino acid residues in the MP06 peptide [32,33]. Although we showed the anticancer activity of MP06 in NSCLC, further studies using its modified and truncated version can optimize the anticancer effect of this ACP.

As most cancer-related deaths are associated with metastasis, the suppression of cancer invasion and migration should be prioritized in anti-cancer therapies [13]. MP06 remarkably inhibited the invasion and migration of all the tested lung cancer cells. EMT is a cellular process involved in the malignant progression of cancer, which primarily regulates intercellular adhesion, cell polarity, and motility [34]. The major EMT regulators are structural proteins (N-cadherin, E-cadherin, and vimentin) and transcription factors (Zeb1, Snail, and Twist); the overexpression of these factors is related to the invasiveness, metastasis, and poor prognosis of NSCLC. The alterations in the expression pattern of structural proteins (N-cadherin and vimentin) drive EMT through a complex network involving various cellular pathways and transcription factors. The expression level of E-cadherin can be considered a tumor suppressor in cases of malignancy and is positively associated with patient survival. Contrastingly, the overexpression of N-cadherin and vimentin is associated with cancer aggressiveness [35]. The lack of vimentin significantly inhibits the migration and deep invasion of cancer cells, whereas its expression is positively correlated with a longer persistence time of migration [36]. Zeb1 expression is accompanied by the downregulation of vimentin, which constrains tumor invasion and migration [37]. Furthermore, the regulation of Snail expression is associated with the expression of membrane proteins (N-cadherin and vimentin) involved in cancer cell adhesion [38]. Immunofluorescence analysis revealed suppressed vimentin expression in NSCLC cell lines treated with MP06, which is consistent with the inhibited expression levels in MP06-treated lung cancer cell lines. Here, we demonstrated that MP06 affects the transcription factors associated with EMT and affects vimentin expression, leading to inhibited cancer cell growth, invasion, and migration. Moreover, MP06 upregulates E-cadherin and downregulates N-cadherin, which may be a candidate inhibitory mechanism of the EMT pathway. Comparative analysis using the EMT-related markers revealed similar suppression of migration and invasion of NSCLC cells treated with MP06, which is consistent with decreased metastasis in a zebrafish model. Zebrafish xenografts, the best model for drug screening in cancer research and preclinical experiments, are a cost-effective and specific tool for exploring cancer therapies [39,40]. Considering the cytotoxicity of MP06 against normal fibroblast cell lines (MRC-5), the minimum concentration (˂20 μM) of MP06 applicable in other experiments was determined based on the cell viability results, indicating its non-cytotoxic concentration. In contrast to cellular assays, the zebrafish model test revealed a 50% lethal toxicity of 4 μM MP06, which indicates that zebrafish embryogenesis, which involves complex and diverse embryological developmental processes, is sensitive to MP06 treatment. However, MP06 significantly reduced metastatic dissemination in zebrafish, which reflects the role of ACP as a potent anti-metastatic agent.

Although the mechanisms underlying the anticancer activity of MP06 are unclear, this molecule effectively inhibited colony formation and proliferation of cancer cells. ERK, which is common in mesenchymal cells, is a potent regulator of EMT, which enhances the migration and infiltration capacities of glioblastoma cancers. Apoptosis is a general mechanism that supports the removal of unwanted cells from the body; it plays a protective role against tumors [41]. Experimental evidence has validated the correlation of apoptosis with the effectiveness of various anticancer drugs and other chemical substances [42]. Upregulated ERK is associated with the inhibition of apoptosis in response to various stimuli, including chemotherapeutic agents [17]. Our findings revealed that MP06 regulates ERK signaling and utilizes this axis to promote EMT, cell migration, and cell invasion at the expense of cell proliferation. Considering the physiological and clinical importance of ERK signaling and the significance of EMT in the development, tissue repair, and progression of cancer, we not only explored previously undescribed connections between ERK and EMT but also identified additional potential therapeutic options for the treatment of aggressive cancers. The interaction between vimentin and ERK serves as a scaffold to recruit Slug to ERK and promote the phosphorylation of Slug. EMT and apoptosis are influenced by the ERK pathway in lung cancer cells [43]. Based on our results, it could be concluded that MP06 could induce apoptosis, but not necrosis, in lung cancer cell lines. The anticancer activity of MP06 was detected to be correlated with the inhibition of the EMT pathway and induction of apoptosis. Furthermore, we verified the anticancer activity of MP06 in vivo using zebrafish xenografts of lung cancer cells. Therefore, MP06 can be considered a novel candidate for the development of anti-cancer therapeutics.

## 4. Materials and Methods

### 4.1. Peptides Design, Synthesis and Purification

The cDNA sequence of *Bryopsis plumosa* was obtained using a PacBio sequencing, single-molecule long-read sequencing method from DNALINK (Seoul, Republic of Korea). Total RNA and cDNA were prepared as previously described and used for cDNA sequencing. The possible anticancer peptide (ACP) was predicted using a custom database which was prepared with the peptide sequence information from the CancerPPD (http://crdd.osdd.net/raghava/cancerppd, accessed on 12 April 2022), CRI database (cancerresearch.org/peptide-database) and AntiCP 2.0 (http://webs.iiitd.edu.in/raghave/anticp2, accessed on 12 April 2022). The peptides that consisted of more than 60 amino acids were removed and small peptides were used for prediction of ACPs. The peptides which had more than 90% sequence similarity were considered as an ACP. Among the ACP candidates, the best anticancer peptides, which were verified sequences of 22 amino acids (LAVISWKCQEWNSLWKKRKRKT) using the ACP model, were used for the anticancer assay. The peptide was synthesized using solid-phase peptide synthesis (AnyGen, Gwangju, Republic of Korea). Peptide was isolated using HPLC on a reversed-phase Vydac C18 column (4.6 mm × 250 mm) equilibrated with 10% acetonitrile solution in 0.05% TFA on an analytical reverse phase high-performance liquid chromatography (Shimadzu, Kyoto, Japan). Peptide elution was carried out using a gradient of acetonitrile concentration (with 0.05% TFA and at a flow rate of 1.5 mL/min): 5% for 30 min, then 10–90% for 60 min (Supplementary Figure S1). A stock solution (10 mM) of the peptide was prepared by dissolving in distilled water and stored at −20 °C until further use.

### 4.2. Cell Culture and Cytotoxic Assay

Human lung cancer cell lines (A549, H460 and H1299) and lung fibroblast MRC-5 cell were purchased from the Korea Cell Line Bank. A549 is an epithelial cell line derived from lung adenocarcinoma. H460 is established from the pleural effusion of a patient with large cell carcinoma. H1299 is lung carcinoma derived from the lymph node. These lung cancer cell lines are widely used for studying various types of tumor progression and drug resistance.

Cells were cultured as monolayer with RPMI-1640 (Hyclone, Cytiva, Marlborough, MA, USA) or DMEM/HIGH GLUCOSE supplemented with 10% fetal bovine serum (FBS) and 1% penicillin/streptomycin (Hyclone). Cells were incubated at 37 °C in a humidified atmosphere with 5% CO_2_. The ability of the peptide to confirm a cytotoxic effect was evaluated using the CCK-8 assay. Cells (5 × 10^3^) were seeded to 96-well plates. The experimental group treated MP06 at different concentrations with culture medium. After the peptide exposure, cells were incubated in a fresh culture medium containing CCK-8 solution for 3 h. Cell viability was determined by measuring absorbance at 450 nm using spectramax i3x (Molecular Devices, San Jose, CA, USA).

### 4.3. Colony-Forming Assay

Each cell was seeded in 35 mm culture dishes at a density of 3000 cells per plate and allowed to attach overnight. Cells were treated with 10 μM peptide. After being incubated for 10 days, cells were stained with 0.5% crystal violet and washed three times with phosphate-buffered saline (PBS). Colonies, defined as groups of ≥50 cells, were counted. Colony-forming units were expressed as a percentage relative to the untreated controls.

### 4.4. Reverse Transcription PCR (RT-PCR)

Total RNA was isolated by scrapping cells with TRIzol reagent as the manufacturer’s instructions (Invitrogen, Carlsbad, CA, USA). First-strand cDNA was synthesized from RNA using a cDNA synthesis kit (iNtRON Biotechnology, Seongnam, Republic of Korea). Resultant cDNA served as templates for PCR amplification with specific primers (Supplementary Table S2). PCR conditions were as follows: initial denaturation at 94 °C for 5 min, 30 cycles of 94 °C for 1 min, 59 °C for 1 min, and 72 °C for 90 s, and a final extension at 72 °C for 10 min. The amplicons were analyzed on 1% agarose gels (iNtRON Biotechnology) using ethidium bromide.

### 4.5. Western Blot Analysis and Immunofluorescence

Lung cancer cells were lysed in RIPA lysis buffer with phosphatase and protease inhibitors cocktails (Sigma Aldrich, St Louis, MO, USA). Protein concentrations were determined via the Bradford assay (Bio-Rad, Hercules, CA, USA). Equal amounts of protein were separated on sodium dodecyl sulphate polyacrylamide gels and transferred to nitrocellulose membranes (Amersham Pharmacia, Pittsburgh, PA, USA). Transferred membranes were incubated with tris-buffered saline (TBS) buffer with 0.1% Tween 20 containing specific antibodies overnight in a cold chamber. After washing with TBS three times, membranes were incubated with mouse secondary antibody (Abcam, Cambridge, MA, USA) and visualized using a Supersignal west atto ultimate sensitivity substrate (Thermo Scientific, Waltham, MA, USA; A38555). Antibodies including N-cadherin (59987), ZEB1 (515797), Vimentin (6260), Snai1 (271977), AKT (5298), p-AKT (271966) and β-actin (47778) were purchased from Santa Cruz Biotechnology. p-ERK (ab278538) and ERK (ab201015) were obtained from Abcam. For immunofluorescence assay, cells were grown onto cover glass in 6-well plates and fixed with 4% paraformaldehyde for 30 min at room temperature. Fixed cells were incubated with antibodies in a solution of PBS at 4 °C overnight. The antibodies used were Vimentin and p-ERK and were visualized using Alexa Fluor 488–conjugated anti-rabbit IgG antibody (Invitrogen, Waltham, MA, USA). Nuclei were counterstained using 4,6-diamidino-2-phenylindole (DAPI; Sigma Aldrich, Burlington, VT, USA). Stained cells were analyzed using a Zeiss LSM510 Meta microscope (Carl Zeiss Micro Imaging GmbH, Göttingen, Germany).

### 4.6. Invasion and Migration Assay

For migration assay, the lower culture chamber of a 24-transwell plate (Cell Biolabs, San Diego, CA, USA) was filled with medium containing RPMI-1640 with 10% FBS (500 µL). Cells (2 × 10^5^) were inoculated in the upper chamber with 200 µL of serum-free medium and incubated at 37 °C for 24 h. To assess invasion, upper chambers used pre-coated with Matrigel (8 μm pores; BD Biosciences, Bedford, MA, USA). Cells were suspended in serum-free medium and placed in the upper invasion chamber. Medium containing 10% FBS was added to the lower chamber. Plates were incubated at 37 °C for 48 h. After removing non-invasive cells from the upper chamber, invasive cells in the lower chamber were fixed with 4% formaldehyde in PBS and stained with crystal violet in ethanol. Matrigel-penetrating cells were counted under a light microscope.

### 4.7. Annexin V/PI Double Staining

The apoptosis assay was performed as previously described [21]. Cells (1 × 10^6^) were cultured with the indicated concentrations of peptide for 72 h. Cells were then washed with PBS and collected via centrifugation. Cells were stained in an Annexin V and propidium iodide (PI) solution (556547; BD Biosciences, San Jose, CA, UAS), followed by incubation at 37 °C for 15 min. Apoptotic cells were analyzed using a flow cytometer (Accuri C6 Plus; BD Biosciences).

### 4.8. In Vivo Test of Zebrafish Embryos and Xenograft

The toxicity of MP06 peptide was confirmed using wild-type zebrafish embryos. Fertilized zebrafish embryos without deficiencies were transferred to a 24-well plate (10 specimens per well). Embryos were exposed to 1, 2, 4, and 10 μM MP06 peptide by dissolving in distilled water. The zebrafish embryos were incubated at 28.5 °C for 48 h and then observed using a stereomicroscope (S6D, Leica, Wetzlar, Germany). The anticancer effect of peptide on A549 cells was validated using zebrafish xenograft assay according to our previous study [31]. Briefly, A549 cells were labeled with 2 μM CM-DiI (Invitrogen, USA) dye for 10 min at 37 °C and washed three times with PBS. For microinjection of cells into the embryos, zebrafish embryos of 2 days post-fertilization (dpf) were anesthetized in tricaine, positioned on a 1.2% low melting agarose gel, and injected with 200 cells into the middle of the embryonic yolk sac region using a Pneumatic Pico-Pump Injector (World Precision Instruments, Sarasota, FL, USA). After injection, the xenografts were cultured in water with 1 and 2 μM of peptide until day 5 at 34 °C. Stained A549 cells were captured and analyzed using a Zeiss LSM510 Meta microscope (Carl Zeiss Micro Imaging GmbH, Göttingen, Germany).

### 4.9. Statistical Analysis

Each value represents the mean ± standard deviation (SD) of at least three independent experiments. GraphPad Prism 10 (GraphPad Software Inc., La Jolla, CA, USA) was used to evaluate the significance of the data through one-way analysis of variance and paired sample *t*-tests at different time points of each experiment.

## 5. Conclusions

NSCLC, the most frequent lung cancer, is a leading cause of mortality and distant metastasis. The development of new anti-cancer therapies is crucial for effective disease management. This study demonstrates the synthesis and functional characterization of an anti-cancer peptide MP06 derived from *Bryopsis plumosa*. The anti-invasive and apoptotic effects of the MP06 were significantly increased in NSCLC cells. In addition, MP06 effectively suppressed the metastasis of tumor xenografts in zebrafish embryos. We suggest considering MP06 as a novel candidate for the development of new anticancer drugs functioning via the ERK signaling pathway.

## Data Availability

The data presented in this study are available in this article.

## Supplementary Materials

The following supporting information can be downloaded at: https://www.mdpi.com/article/10.3390/md21120607/s1, Figure S1: Chromatographic profiles(A) and mass spectra(B) for MP06 (theoretical 2788.18 Da); Figure S2: Screening of the anticancer peptide; Table S1: The list of predicted ACPs using the database; Table S2: Primer sequences for RT-PCR.
