# Supplementary material for: A Novel Anticancer Peptide Derived from Bryopsis plumosa Regulates Proliferation and Invasion in Non-Small Cell Lung Cancer Cells
Source: Mar Drugs. 2023 Nov 24;21(12):607. doi: 10.3390/md21120607 (PMC10744475; doi:10.3390/md21120607)
Supplement: Supplementary file 1 [file marinedrugs-21-00607-s001.zip › marinedrugs-2688278-supplementary.pdf]

Supplementary data

A novel anticancer peptide derived from *Bryopsis plumosa* regulates proliferation and invasion in Non-Small Cell Lung Cancer cells

Heabin Kim[1,†], Hyun-Taek Kim[2,†] Seung-Hyun Jung[1], Jong Won Han[1], Seonmi Jo[1], In-Gyu Kim[3], Rae-Kwon Kim[3, 4], Yeon-Jee Kahm[3,4], Tae-Ik Choi[5], Cheol-Hee Kim[5] and Jei Ha Lee[1,*]

[1] Department of Genetic Resources, National Marine Biodiversity Institute of Korea, Seocheon 33662, Republic of Korea.
[2] Soonchunhyang Institute of Medi-bio Science (SIMS), Soonchunhyang University, 31151 Cheonan-si, Republic of Korea.
[3] Department of Radiation Biology, Environmental Safety Assessment Research Division, Korea Atomic Energy Research Institute, Daejeon 34057, Republic of Korea.
[4] Department of Radiation Science and Technology, Korea University of Science and Technology, Daejeon 34113, Republic of Korea
[5] Department of Biology, Chungnam National University, Yuseong-gu, Daejeon 34134, Republic of Korea

*Correspondence: jeiha@mabik.re.kr;
E-mail: jeiha@mabik.re.kr

2 Figures, 2 Tables, Statistical Data

Supplementary Figure S1

A

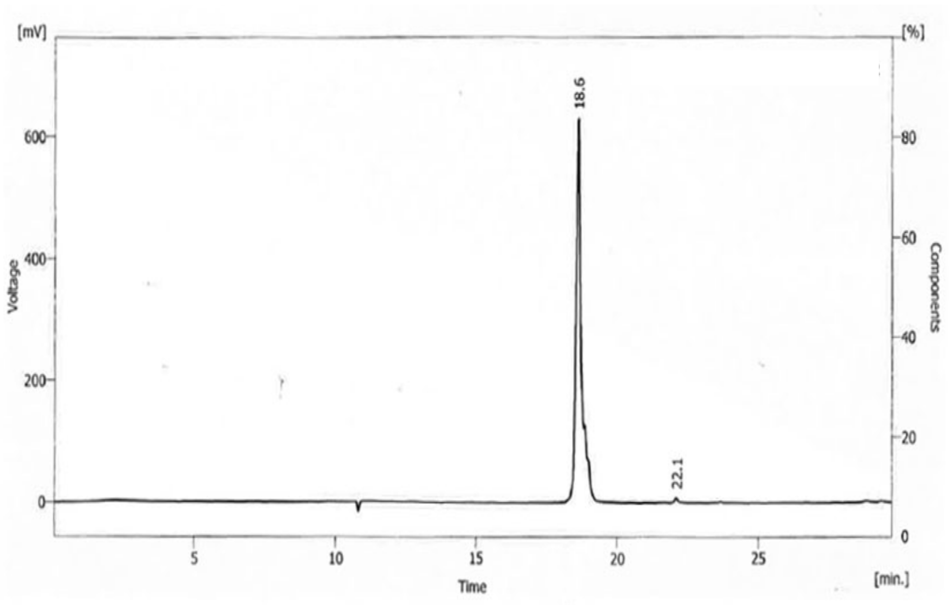

B

MP06

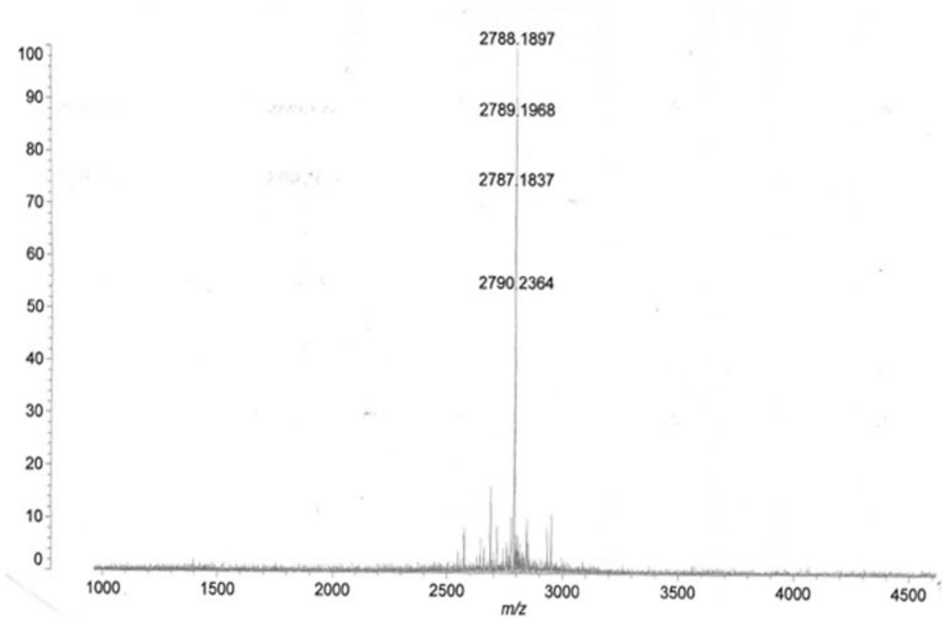

Figure S1. Chromatographic profiles(A) and mass spectra(B) for MP06 (theoretical 2788.18 Da). The peptide (LAVISWKCQEWNSLWKKRKRKT) was obtained using solid-phase peptide synthesis.

Supplementary Figure S2

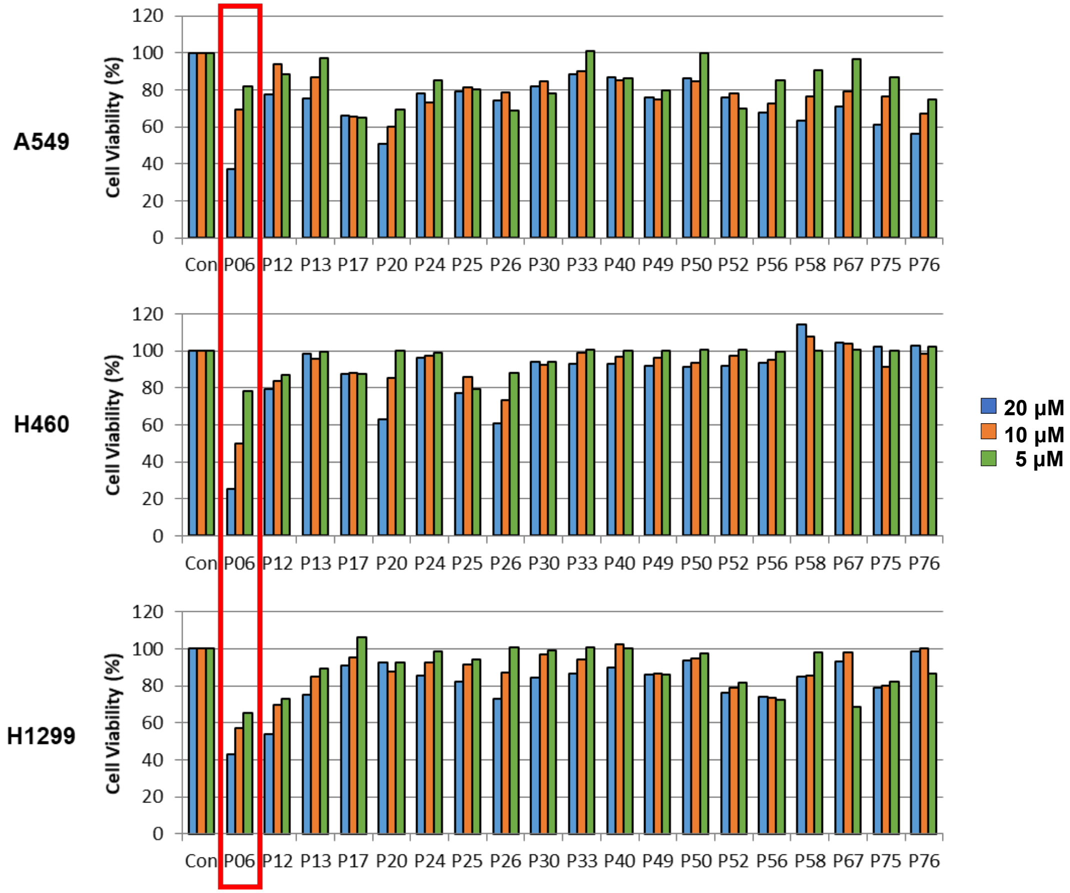

Figure S2. Screening of the anticancer peptide. Effect of predicted ACPs at a concentration of 20, 10 and 5 μM on the proliferation of NSCLCs for 48 h.

Supplementary Table S1

Table S1. The list of predicted ACPs using the database

Name	Query Pep Seq	Model1 prediction	Model1 score	Model2 prediction	Model2 score	AA number
MP06	LAVISWKCQEWNSLWKKRKRKT	Non-AntiCP	0.42	AntiCP	0.57	22
MP12	GSSSWLPTFRHLFGCHSPEMTSHLLR	AntiCP	0.55	Non-AntiCP	0.35	26
MP13	QDFFVWSKCIQVRMGCPQRQKICW	AntiCP	0.49	AntiCP	0.52	24
MP17	MEVTFGENLNPSLLRRMRRKLAGVELSEVR	AntiCP	0.48	Non-AntiCP	0.08	30
MP20	MLWLHFFPLFQKIPFNIRNGFIGMFCSFKFMFKRKTFSWTMEK	AntiCP	0.5	Non-AntiCP	0.43	43
MP24	PVSRSAKAGLQFPVGRIARYLKKGK	Non-AntiCP	0.4	AntiCP	0.68	25
MP25	SFPAPTKPLKVVVIGAGLAGLSAAK	Non-AntiCP	0.43	AntiCP	0.85	25
MP26	APYTTGEWKGCNVSCGAGVENR	Non-AntiCP	0.27	AntiCP	0.55	22
MP30	LFLLLSMTMVLGAAAPKDEFIDIEPVQK	AntiCP	0.51	Non-AntiCP	0.1	28
MP33	LPGSRPKGESCCFTPAIF	Non-AntiCP	0.35	AntiCP	0.66	18
MP38	SERFFFFPECVLCVCLGSGEWGETK	Non-AntiCP	0.41	AntiCP	0.48	25
MP40	QMTRSAKAGLQFPVGRIARYLKK	Non-AntiCP	0.43	AntiCP	0.52	23
MP49	LDHQTVAKFMGIVISALGK	Non-AntiCP	0.27	AntiCP	0.45	19
MP50	APRWSPARRAGHWWGRPLPDHTLPPPSCGTPPSC	AntiCP	0.56	AntiCP	0.56	34
MP52	MSCTHLGSTRRHLSPSLKCRCGGVS	Non-AntiCP	0.36	AntiCP	0.54	25
MP56	DLPGRPDIAYIGRRRAIFVHGCFWHGHDCK	AntiCP	0.46	Non-AntiCP	0.37	30
MP58	VYFNGCLPHQFCSSNKLLCNFLPLPLFH	AntiCP	0.5	AntiCP	0.47	28
MP67	PGTGLAHKACANLCLIGG	AntiCP	0.46	AntiCP	0.73	18
MP69	MVIGAWCGASVRFFLVGCQVGC	AntiCP	0.5	AntiCP	0.58	22
MP75	MTPYMRAAIACAALVALAAL	AntiCP	0.64	Non-AntiCP	0.26	20
MP76	AIACAALVALAALGDARPVV	AntiCP	0.5	Non-AntiCP	0.24	20

Supplementary Table S2

Table S2. Primer sequences for RT-PCR

Primer name	Sequence
N-cad-fw	ACTTGCCAGAAAACTCCAGG
N-cad-rv	TGGTGTATGGGGTTGATCCT
E-cad-fw	TGGATAGAGAACGCATTGCC
E-cad-rv	AAAATCCAAGCCCGTGGTG
Vimentin-fw	GAGAACTTTGCCGTTGAAGC
Vimentin-rv	TCTGCTGGTATATGAGTGCTG
Zeb1-fw	CGGCGCAATAACGTTACAAA
Zeb1-rv	AAAGGTGTAACTGCACAGGG
Snai1-fw	GGGACTGTGAGTAATGGCTG
Snai1-rv	CCCACTCCTCTATGACACCA
P53-fw	ATGTTTTGCCAACTGGCCAAG
P53-rv	TGAGCAGCGCTCATGGTG
Bax-fw	ATGTTTTCTGACGGCAACTTC
Bax-rv	AGTCCAATGTCCAGCCCAT
Caspase3-fw	TGTTTGTGTGCTTCTGAGCC
Caspase3-rv	CACGCCATGTCATCATCAAC
GAPDH-fw	GACAGTCAGCCGCATCTTCT
GAPDH-rv	GCGCCCAATACGACCAAATC

Statistical Data

Figure 2. B

MRC-5	Contr	20 µM	10 µM	5 µM		A549	Contr	20 µM	10 µM	5 µM
1	0.687	0.412	0.543	0.677		1	2.16	0.541	1.109	1.852
2	0.623	0.396	0.497	0.669		2	2.05	0.518	1.146	1.647
3	0.641	0.331	0.563	0.656		3	2.15	0.496	1.011	1.549
Mean	0.65	0.379667	0.534333	0.667333		Mean	2.12	0.518333	1.088667	1.682667
SD	0.03	0.04	0.033843	0.010599		SD	0.06	0.02	0.069759	0.154617
p-value		0.000978	0.008028	0.002897		p-value		1.79E-06	0.000175	0.003731
H460	Contr	20 µM	10 µM	5 µM		H1299	Contr	20 µM	10 µM	5 µM
1	1.02	0.389	0.6415	0.869		1	0.312	0.104	0.157	0.236
2	1.04	0.357	0.525	0.932		2	0.362	0.103	0.162	0.252
3	1.01	0.316	0.544	0.911		3	0.331	0.119	0.179	0.267
Mean	1.02	0.354	0.570167	0.904		Mean	0.34	0.108667	0.166	0.251667
SD	0.02	0.04	0.062503	0.032078		SD	0.03	0.01	0.011533	0.015503
p-value		8.15E-06	0.006654	0.001188		p-value		0.000127	0.002445	0.001546

Figure 2. D

MRC-5	Contr	10 µM		A549	Contr	10 µM
1	213	215		1	642	237
2	264	206		2	598	221
3	234	234		3	651	187
Mean	237.00	218.3333		Mean	630.33	215
SD	25.63	14.29		SD	28.36	25.53
p-value		0.332447		p-value		4.66E-05
H460	Contr	10 µM		H1299	Contr	10 µM
1	711	342		1	652	245
2	770	351		2	619	251
3	765	392		3	701	266
Mean	748.67	361.6667		Mean	657.33	254
SD	32.72	26.65		SD	41.26	10.82
p-value		9.18E-05		p-value		8.14E-05

Statistical Data

Figure 3. A

	Migration				Invasion	
A549	Contr	10 μM		A549	Contr	10 μM
1	0.464	0.037		1	0.541	0.17
2	0.451	0.121		2	0.567	0.166
3	0.507	0.087		3	0.607	0.149
Mean	0.47	0.081667		Mean	0.57	0.161667
SD	0.03	0.04		SD	0.03	0.01
p-value		0.000189		p-value		3.51E-05
H460	Contr	10 μM		H460	Contr	10 μM
1	0.327	0.142		1	0.182	0.04
2	0.296	0.151		2	0.183	0.051
3	0.297	0.131		3	0.182	0.067
Mean	0.31	0.141333		Mean	0.18	0.052667
SD	0.02	0.01		SD	0.00	0.01
p-value		0.000146		p-value		7.85E-05
H1299	Contr	10 μM		H1299	Contr	10 μM
1	0.627	0.219		1	0.527	0.142
2	0.626	0.151		2	0.526	0.134
3	0.627	0.148		3	0.527	0.097
Mean	0.63	0.172667		Mean	0.53	0.124333
SD	0.00	0.04		SD	0.00	0.02
p-value		4.01E-05		p-value		8.39E-06

Figure 5. A

Survial Rate of Zebrafish Embryos (%)					
MP06 (μM)					
	Contr	1	2	4	10
1	100	100	90	70	0
2	90	90	90	70	0
3	100	100	80	60	0

Figure 5. B

Metastatic Rate of Zebrafish Embryos (%)			
MP06 (μM)			
	Contr	1	2
1	85.71	66.66	37.5
2	75	62.5	50
3	75	62.5	33.33
